# *EHP* Classic Paper of the Year, 2012

**DOI:** 10.1289/ehp.1205408

**Published:** 2012-07-02

**Authors:** Hugh A. Tilson

**Affiliations:** Editor-in-Chief, *EHP*, E-mail: tilsonha@niehs.nih.gov

*Environmental Health Perspectives* (*EHP*) established the Paper of the Year in 2008 (Tilson 2008) as a way of highlighting high-quality articles published in the journal. The *EHP* Classic Paper of the Year is given to the most highly cited Research Article, Commentary, or Review Article over the preceding 60 months. We are proud to announce that the 2012 *EHP* Classic Paper of the Year is “Exposure of the U.S. Population to Bisphenol A and 4-*tertiary*-Octylphenol: 2003–2004” by Antonia M. Calafat, Xiaoyun Ye, Lee-Yang Wong, John A. Reidy, and the late Larry L. Needham of the Division of Laboratory Sciences, National Center for Environmental Health, Centers for Disease Control and Prevention. This article (Calafat et al. 2008) was published in the January 2008 issue of *EHP*; since its publication it has been cited > 40 times/year.

When the paper was published, public and scientific concerns related to health and exposure to bisphenol A (BPA) had already emerged. BPA, a high production volume chemical, is used worldwide to manufacture many consumer items, such as reusable water bottles and food containers; linings of metal food cans; medical devices and impact-resistant safety equipment; and polyvinyl chloride, paper, and cardboard products. Results from animal studies suggested that exposures to BPA can lead to developmental, reproductive, and other adverse health effects. Yet, important knowledge gaps remained regarding the relevance of these findings to human health, in part, because of limited information on human exposure to BPA. Calafat et al. (2008) addressed this gap using bio-monitoring—the accurate and precise measurement of trace levels of environ-mental chemicals in a person’s body—to investigate the degree of the U.S. general population’s exposures to BPA.

Calafat et al. (2008) used state-of-the-art analytical chemistry methods to measure the urinary levels of BPA in a representative sample of the U.S. population: 2,517 participants > 6 years of age in the 2003–2004 National Health and Nutrition Examination Survey (NHANES). The study demonstrated that almost 93% of the NHANES participants were exposed to BPA. Because BPA is transformed and eliminated from the body within a few hours after exposure, the high percentage of people with detectable BPA suggested frequent or almost continuous exposure of the U.S. general population to this chemical. Furthermore, using urinary levels of BPA to categorize exposures, Calafat et al. (2008) showed that exposure differed by race/ethnicity, age, sex, and household income, suggesting that exposure may be influenced by multiple factors. The authors’ findings on BPA exposure also highlighted the need for additional public health research. Calafat et al. established the framework that led the way for future studies to identify sources and routes of exposure to BPA and to evaluate potential health effects resulting from such exposures. Since the publication of the paper, our knowledge of exposure to BPA has greatly improved. For example, in addition to diet (still believed to be the main contributor to exposure for the general population), we now know that medical interventions (e.g., Calafat et al. 2009) and thermal paper products such as store receipts (e.g., Biedermann et al. 2010) can also contribute to BPA exposure. Moreover, additional high-quality biomonitoring studies of populations exposed to BPA—published since the Calafat et al. (2008) paper—have increased our understanding of the potential links between BPA exposures and human health. This exposure information will be useful for risk assessment (e.g., to set intervention and research priorities and evaluate the effectiveness of public health measures), as well as to monitor exposure trends.

*EHP* congratulates Calafat and colleagues for their contribution to the environmental health science literature. Their work clearly illustrates the utility of human biomonitoring to estimate population-based exposures. These exposure and health effects studies will ultimately inform policy decisions regarding human exposure levels and direct appropriate public health actions.

## Editor's Note: Manuscript Processing Fee

Starting 1 August 2012, *Environmental Health Perspectives* will begin charging a flat fee for processing manuscripts for publication. On acceptance of a manuscript, authors will be required to pay $750, $1,500, or $1,000 for a Commentary, Review Article, or Research Article, respectively. An additional $500 will be charged for Supplemental Material exceeding 2,000 words, which includes tables, figure legends, text, and references. Each figure counts as 250 words. The manuscript processing fee replaces the page charge of $35 per accepted Microsoft Word manuscript page and charges for color figures. Word limits for Commentaries, Review Articles, and Research Articles will remain at 5,000, 10,000, and 7,000 words, respectively.
